# Identifying Plant Pentatricopeptide Repeat Proteins Using a Variable Selection Method

**DOI:** 10.3389/fpls.2021.506681

**Published:** 2021-03-01

**Authors:** Xudong Zhao, Hanxu Wang, Hangyu Li, Yiming Wu, Guohua Wang

**Affiliations:** ^1^College of Information and Computer Engineering, Northeast Forestry University, Harbin, China; ^2^State Key Laboratory of Tree Genetics and Breeding, Northeast Forestry University, Harbin, China

**Keywords:** pentatricopeptide repeat, variable selection, variable importance, random forest, model selection, Gaussian mixture model

## Abstract

**Motivation:** Pentatricopeptide repeat (PPR), which is a triangular pentapeptide repeat domain, plays an important role in plant growth. Features extracted from sequences are applicable to PPR protein identification using certain classification methods. However, which components of a multidimensional feature (namely variables) are more effective for protein discrimination has never been discussed. Therefore, we seek to select variables from a multidimensional feature for identifying PPR proteins.

**Method:** A framework of variable selection for identifying PPR proteins is proposed. Samples representing PPR positive proteins and negative ones are equally split into a training and a testing set. Variable importance is regarded as scores derived from an iteration of resampling, training, and scoring step on the training set. A model selection method based on Gaussian mixture model is applied to automatic choice of variables which are effective to identify PPR proteins. Measurements are used on the testing set to show the effectiveness of the selected variables.

**Results:** Certain variables other than the multidimensional feature they belong to do work for discrimination between PPR positive proteins and those negative ones. In addition, the content of methionine may play an important role in predicting PPR proteins.

## 1. Introduction

Pentatricopeptide repeat (PPR), which is a 35-amino acid sequence motif (Chen et al., 2018; Rojas et al., 2018) and is commonly found in eukaryotes and terrestrial plants (Ruida et al., 2013), plays an important role in plant growth and development (Qu et al., 2019). PPR proteins, which are distinguished by the presence of tandem degenerate PPR motifs and by the relative lack of introns in the genes coding for them, are regarded as an ideal model to study plant cytoplasmic and nuclear interactions (Wang et al., 2008).

Many prevailing methods or tools (Wei et al., 2017a,b; Tang et al., 2018) can be used to predict PPR proteins. A feature composed of 188 variables (namely 188D) related to sequence information and amino acid properties (Zhang et al., 2012; Song et al., 2014; Xu et al., 2014; Li et al., 2019) or the one including 65 components, i.e., pseudo-amino acid composition which can be abbreviated as PAAC (Chou, 2001, 2005) is a case in point. In addition, classifiers such as random forest (Lv et al., 2019; Ru et al., 2019; Wei et al., 2019) and support vector machine (Tang et al., 2016; Tan et al., 2019) can be applied to the evaluation of extracted features. Commonly, these features represent the content of certain amino acid, the conversion frequency of its surface tension, its hydrophobicity, hydrophilicity, and side chain volume, etc. However, it has never been discussed whether only some components of a multidimensional feature may work or not. In other words, which components of an extracted feature may identify PPR proteins (i.e., distinguish PPR proteins from non-PPR ones) need to be discussed.

**Figure 1:**
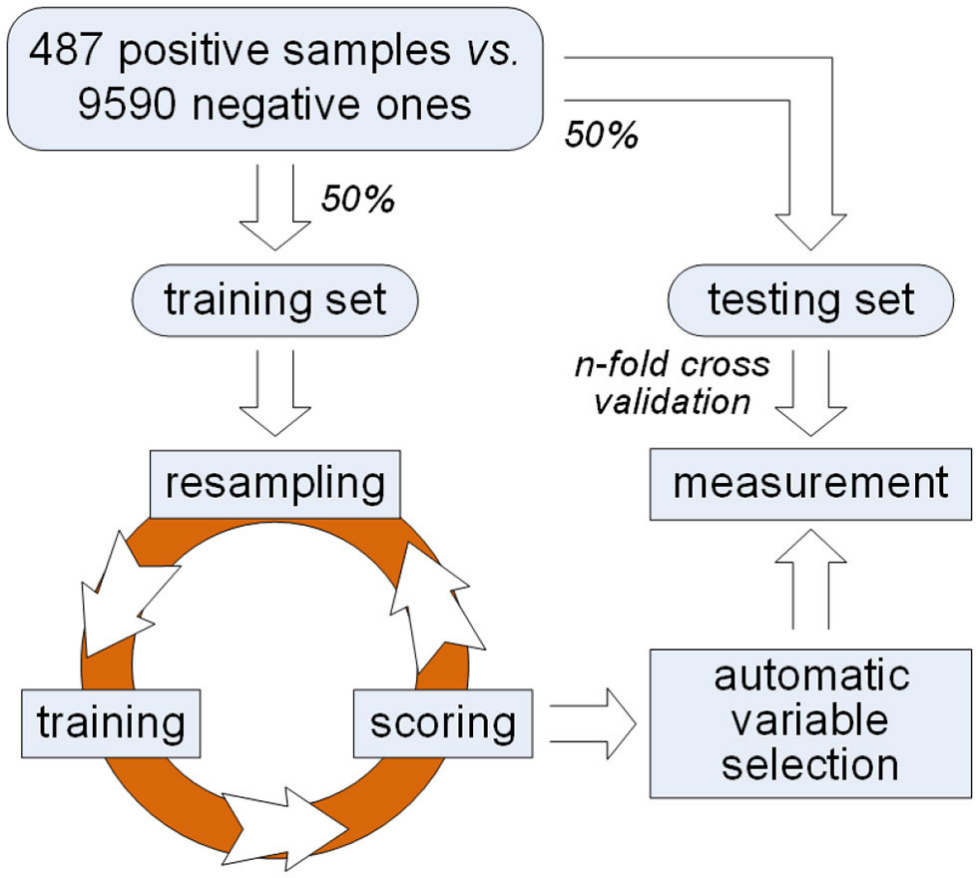
A framework of variable selection for identifying plant PPR proteins.

In order to solve this problem, we propose a framework of variable selection for identifying plant PPR proteins as shown in Figure 1. First of all, samples are randomly split in balance within either PPR positive or negative protein group. Then, multiple rounds of resampling, training and scoring are implemented on the training set in order to accumulate scores for each variable. Random forest is presented as the ensemble classifier to be trained. In each round, the score of a variable is calculated by making a comparison between classification error rates before and after one time random permutation of the remaining sample values on the variable. After enough rounds of score accumulation, variables with high accumulated scores are regarded as important variables. Instead of manually choosing variables with high accumulated scores, we make an automatic variable selection according to their accumulated scores by model selection based on Gaussian mixture model. After important variables are selected, qualitative and quantitative measurements are made on the testing set derived from previous sample split. Good classification results indicate the effectiveness of the selected variables which keep certain properties for identifying PPR proteins.

## 2. Method

First of all, the dataset representing plant PPR is provided (Qu et al., 2019), which contains 487 PPR positive and 9,590 negative protein primary sequences. Subsequently, features including 188D and PAAC are extracted, respectively. Commonly, these features are directly used for distinguishing positive proteins from negative ones. However, which components of 188D or PAAC do play a part in this discrimination needs to be further discussed. Thus, we follow the framework presented in Figure 1 to select key variables for identifying plant PPR proteins. More details can be seen in the following subsections.

### 2.1. Sample Split

In order to validate the effectiveness of selected variables, we make a balanced sample split. Samples within positive or negative group are equally divided other than splitting negative samples in 10 sets (Qu et al., 2019), which actually discarded half negative samples. As a result, 243 positive samples and 4,795 negative ones are randomly selected as a training set; meanwhile, the remaining samples are regarded as a testing set.

### 2.2. Resampling, Training, and Scoring

As illustrated in Figure 1, an iteration is implemented on the training set for obtaining important variables. Each round of the iteration includes three steps, i.e., resampling, training, and scoring. First of all, we randomly choose two-thirds of the training samples in balance. That is, 162 positive samples and 3,197 negative ones are selected in a random manner for the next training step.

Secondly, these selected samples are utilized to train a classification and regression tree (CART). All the components of a feature are considered. By recursively splitting data into distinct subsets, the CART is constructed in a binary-tree form. At each node of the CART, Gini impurity (GI) is used to choose a variable. In fact, GI is a measure of how often a randomly chosen sample point from training set would be incorrectly labeled. It can be computed by summing all the probability products, each of which is expressed as a probability of a randomly chosen sample labeled *i* (i.e., *p*_*i*_) times the probability 1 − *p*_*i*_. That is,

(1) $$GI=\sum_{i=0}^{k}p_{i}*(1-p_{i}),$$

where *k* is the number of sample groups in the training set and *k* = 2. To select suitable variable to make a split in each node, the decrease of GI between the parent node and its two descendant nodes is calculated, and the variable *m* which maximizes this decrease is chosen as the current node.

Thirdly, the remaining one-third of the training samples, which are also named as out-of-bag (OOB) samples, are used for scoring variable importance. At the scoring step, we adopt a permutation based variable importance scoring approach. The main idea behind this method is that we use the classifier to predict labels of OOB samples and calculate the classification accuracy or error rate in advance. In our experiments, OOB error estimate is utilized. The established CART is used to classify each OOB sample. Taking the unbalanced distribution between positive and negative samples into account, we modify the OOB error rate as follows,

(2) $$Err_{OOB}=(FN/(TP+FN)+FP/(TN+FP))/2,$$

where *FN*, *TP*, *FP*, and *TN* represent the number of false negative, true positive, false positive, and true negative samples, respectively. Then we permute the values of a specific variable and use the classifier to predict the permuted data and calculate the error rate again. The difference between the two error rate measures is assigned to the specific variable as its importance.

Under the assumption that there are no differential expression levels between positive and negative samples, the expression levels of OOB samples on component *i* are reordered. Correspondingly, a new OOB classification error rate which is expressed as ${\tilde{Err}}_{OOB}$ is obtained using Equation (2). As a result, the score of component *i* in *j* round is calculated as

(3) $$score_{j}(i)={\tilde{Err}}_{OOB}-Err_{OOB}.$$

The score calculated in Equation (3) indicates the contribution of component *i* to the classification result expressed in Equation (2). If the values of variable *i* have no apparent difference between two groups of OOB samples before and after permutation, then ${\tilde{Err}}_{OOB}$ will keep a similar OOB error rate as *Err*_*OOB*_ despite the one time permutation to the values of OOB samples. Otherwise, the score expressed in Equation (3) will become large. After *N* rounds of resampling, training and scoring, the accumulated score of component *i* is expressed as

(4) $$Acc\_score\left(i\right)=\frac{\sum_{j=1}^{N}score_{j}\left(i\right)}{N}.$$

### 2.3. Automatic Variable Selection

Once the accumulated score of each component or variable in a multidimensional feature is achieved, it needs to be further discussed either some components or the whole feature may work for discrimination between positive and negative samples. Instead of manually selecting variables with high accumulated scores, a model selection method needs to be presented for automatic variable selection. Here, we choose Gaussian mixture model (GMM) (Li et al., 2015) for automatic variable selection.

GMM is a probabilistic model that assumes samples are generated from the mixture of Gaussian distributions. As a result, data is distributed as follows,

(5) $$\begin{array}{r} p\left(x|\theta \right)=\overset{k}{\underset{i=1}{\sum }}\pi_{i}N\left(\mu_{i},\sigma_{i}\right), \end{array}$$

where *π*_*i*_, *μ*_*i*_, *σ*_*i*_ are the mixture proportion, the mean and the standard variance of Guassian component *i*, respectively. *N* denotes Gaussian distribution, and *θ* = (*π*_*i*_, *μ*_*i*_, *σ*_*i*_) is the parameter vector to be determined. To fit a GMM model, expectation maximum algorithm (EM) which guarantees converge can be used after fixing the number of components. EM algorithm is an iterative method to find the maximum likelihood, or maximum posteriori estimates on parameters of a model. The method repeatedly performs the expectation (E) step and the maximum (M) step. In the E step, a function for the expectation of the log-likelihood evaluated using the current estimates for model parameters is created; while in the M step, the values of parameters which maximize the function for the expectation is found, and the new estimates are then used in the next E step. When fitting the GMM model, the E step at the *i*th iteration of EM algorithm can be described as

(6) $$\begin{array}{c} p^{i}=\frac{\pi p(x\text{|}\mu ,\sigma )}{\sum_{k}\pi p(x\text{|}\mu ,\sigma )}. \end{array}$$

And the updates of estimates at the (*i* + 1)th iteration in the M step is formulated as

(7) $$\begin{array}{l} \pi^{i+1}=\frac{\sum p^{i}}{N},\\ \mu^{i+1}=\frac{\sum p^{i}x}{\sum p^{i}},\\ \sigma^{i+1}=\frac{\sum p^{i}(x-\mu^{i}){\left(x-\mu^{i}\right)}^{T}}{\sum p^{i}}. \end{array}$$

How to choose the GMM model which fits the data best among these with different component numbers is an instance of model selection. Bayesian information criterion (BIC) is employed in our method. BIC is defined as

(8) $$BIC=ln(n)k-2ln(\hat{L}),$$

where $\hat{L}=p\left(x\text{|}\hat{\theta },\hat{L}\right)$ denotes the maximized value of the likelihood function of GMM, *n* is the sample size and *k* is the number of parameters in the model. BIC considers both data fitting and model complexity, and it adds a penalty term for each model to help to avoid overfitting. The model with the lowest BIC is preferred.

After fitting the GMM model with accumulated scores, variables belonging to Gaussian distributions with high means will be automatically selected for subsequent analysis.

### 2.4. Measurement

In order to show the effectiveness of the selected variables, we choose seven quantitative measurements including confusion matrix, TP rate, FP rate, Precision, Recall, Accuracy, and F1-measure.

A confusion matrix (Theodoridis and Koutroumbas, 2009) illustrates the number of false negative (FN), true positive (TP), false positive (FP), and true negative (TN) samples. Correspondingly, TP rate, FP rate, Precision, Recall, and Accuracy (ACC) are computed as follows,

(9) $$\begin{array}{l} TP\;rate=\frac{TP}{TP+FN},\\ FP\;rate=\frac{FP}{FP+TN},\\ Precision=\frac{TP}{TP+FP},\\ Recall=\frac{TP}{TP+FN},\\ ACC=\frac{TP+TN}{TP+FN+TN+FP}, \end{array}$$

where TP rate and Recall are expressed in the same form. The F1-measure (Nan et al., 2012) is a harmonic average of Precision and Recall, which is expressed as

(10) $$F1-measure=\frac{2*Precision\;*\;Recall}{Precision+Recall}.$$

Besides, the receiver operating characteristic (ROC) and the area under ROC curve (AUC) are also provided.

## 3. Results

Experiments were conducted on 487 PPR positive and 9,590 negative proteins. The procedure shown in Figure 1 was accomplished using our own developed tool ECFS-DEA (Zhao et al., 2020) on the training set, whereby variables associated with discrimination between PPR positive and negative samples were automatically selected. On the testing set, we used six-fold cross validation. Five parts of the testing set were used to train a random forest (RF), each tree of which was a CART. The remaining part was used for testing.

### 3.1. Variable Selection Results on 188D

**Figure 2:**
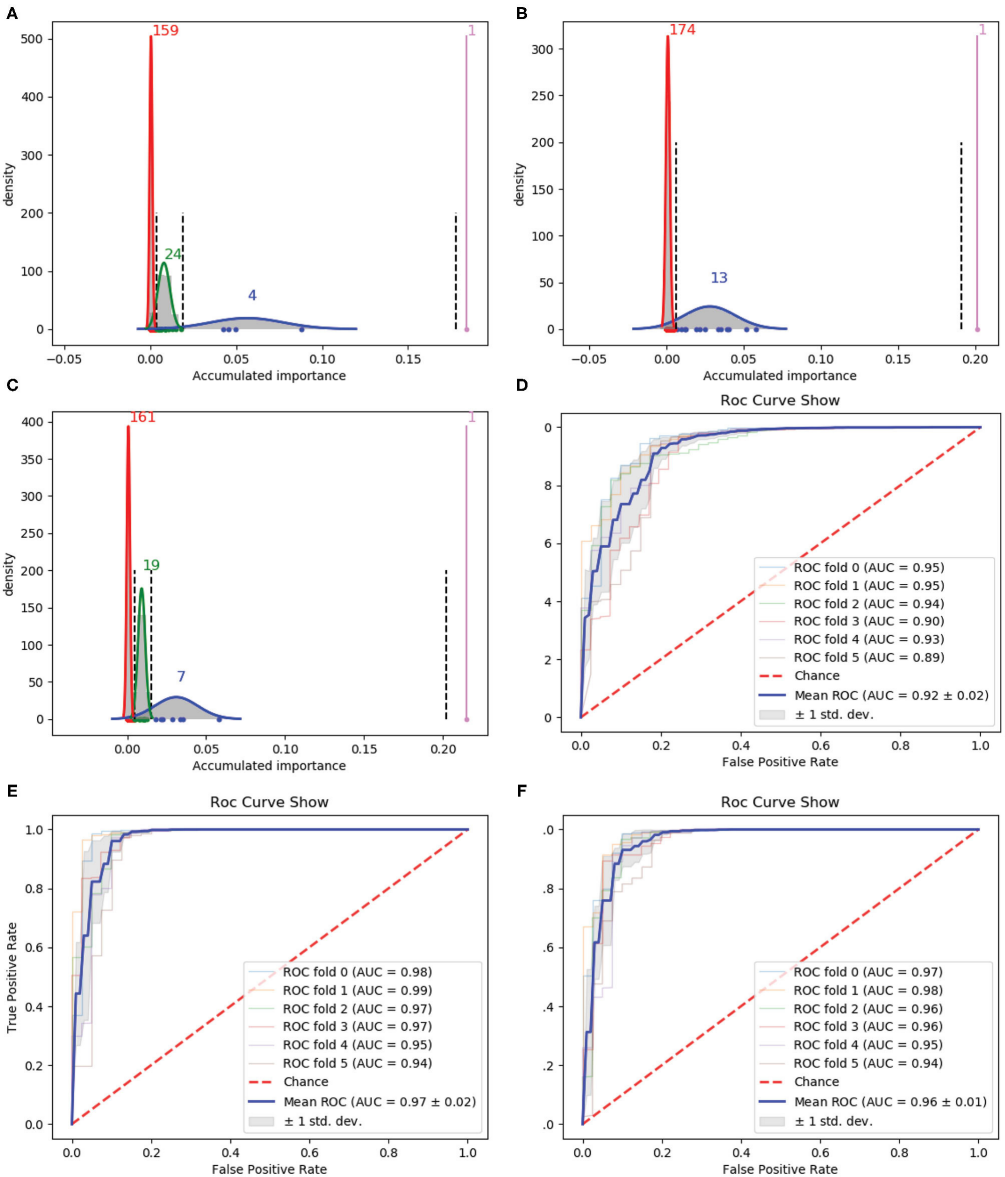
Qualitative results on 188D. **(A–C)** Gaussian distribution of the accumulated variable importance corresponding to three-time random sample splits, respectively. **(D–F)** Refer to the ROC curves and AUC values of the selected four variables, 188D and the selected 13 variables, respectively.

We firstly used 188D as the starting point of our variable selection method. Rounds of the iteration were referred to the successively performing of resampling, training, and scoring. In order to stabilize the results obtained by our variable selection method, 1 × 10^5^ rounds were performed. In addition, this procedure was repeated three times, each of which corresponded to a group of randomly selected training samples. Accordingly, Gaussian distributions of the GMM instances fitted by the accumulated variable importance are listed in Figures 2A–C, respectively.

Figure 2A illustrates one, four, 24 and 159 variables in the first, second, third, and fourth Gaussian component with the variable importance in a descending order, respectively. Figure 2B shows one, 13 and 174 variables in the first, second, and third Gaussian component with the variable importance in a descending order, respectively. As to Figure 2C, it has one, seven, 19 and 161 variables in the first, second, third, and fourth Gaussian component according to the variable importance in a descending order. If we select variables which belong to the first two Gaussian components of all the three times which have highest mean scores of variable importance, namely we select all the variables belong to subset Q, where *Q* = ∩_*i*_(*G*_*i*1_ ∪ *G*_*i*2_). Here, *i* is the index of the three times and *G*_*ij*_ denotes the Gaussian distribution which has the *j*th highest mean score of variable importance in the *i*th time, four variables are selected. The corresponding ROC curves and AUC values can be seen in Figure 2D.

If we expand the scope by including two green Gaussian components illustrated in Figures 2A,C, namely, we now select variables in subset *Q*′, where *Q*′ is defined as $Q^{\prime }=\left(G_{11}\cup G_{12}\cup G_{13}\right)\cap \left(G_{21}\cup G_{22}\right)\cap \left(G_{31}\cup G_{32}\cup G_{33}\right)$, we will obtain 13 variables. The corresponding ROC curves and AUC values can be seen in Figure 2F. Besides, the ROC curves and AUC values of 188D are shown in Figure 2E.

Starting from the set *A* which is composed of the only variable with the highest importance score, i.e., *A* = ∩_*i*_*G*_*i*1_, we progressively add to *A* new elements in *Q* which is made up of originally selected four variables with their importance in a descending order and present quantitative results in Table 1. Detailed results of the selected four variables together with the 13 variables and 188D are also listed in Table 1.

**Table 1:** Quantitative results on 188D.

**Feature**	**Confusion matrix**			**Class**	**TP rate**	**FP rate**	**Precision**	**Recall**	**F1-measure**
(10)^*T*^	Classified as − >	a	b	a: Positive	0.230	0.032	0.267	0.230	0.247
	a	56	188	b: Positive	0.968	0.770	0.964	0.968	0.964
	b	154	4,641	Weighted average	0.932	0.734	0.927	0.932	0.927
(10, 12)^*T*^	Classified as − >	a	b	a: Positive	0.344	0.012	0.587	0.344	0.434
	a	84	160	b: Positive	0.988	0.656	0.967	0.988	0.977
	b	59	4,736	Weighted average	0.957	0.625	0.949	0.957	0.951
(10, 12, 130)^*T*^	Classified as − >	a	b	a: Positive	0.434	0.011	0.675	0.434	0.528
	a	106	138	b: Positive	0.989	0.566	0.972	0.989	0.980
	b	51	4,744	Weighted average	0.962	0.539	0.958	0.962	0.958
(10, 12, 130, 1)^*T*^	Classified as − >	a	b	a: Positive	0.541	0.006	0.825	0.541	0.653
	a	132	112	b: Positive	0.994	0.459	0.977	0.994	0.985
	b	28	4,767	Weighted average	0.972	0.437	0.970	0.972	0.969
(10, 12, 152, 130,	Classified as − >	a	b	a: Positive	0.639	0.001	0.975	0.639	0.772
1, 63, 24, 13, 22,	a	156	88	b: Positive	0.999	0.361	0.982	0.999	0.990
87, 62, 45, 9)^*T*^	b	4	4,791	Weighted average	0.982	0.344	0.982	0.982	0.979
188D	Classified as − >	a	b	a: Positive	0.623	0.000	1.000	0.623	0.768
	a	152	92	b: Positive	1.000	0.377	0.981	1.000	0.985
	b	0	4,795	Weighted average	0.982	0.359	0.982	0.982	0.979

In Table 1, the confusion matrix, true positive (TP) rate, false positive (FP) rate, precision, recall, and F1 measure are calculated for the results corresponding to a specific feature, i.e., the compound of the selected variables. The two classes representing PPR positive (labeled *a*) and negative proteins (labeled *b*) are separately considered as the positive class when we calculate these quantitative results. As more and more variables are added to *A*, the frequency of misclassifying samples labeled *b* to *a* decreases and vice versa; while the TP rate using both class *a* and class *b* as the positive class improves, so do the precision, recall and F1 measure. As to the FP rate, when setting the positive class to *b* and the error rate that misclassifying samples of label *a* to *b*, the values gradually become smaller as more variables are included at the beginning, but fluctuate later. These dynamic changes are illustrated in **Figures 4A,B**, respectively. Besides, it can be obviously seen that the 13 variables keep a comparable result with 188D.

### 3.2. Variable Selection Results on PAAC

**Figure 3:**
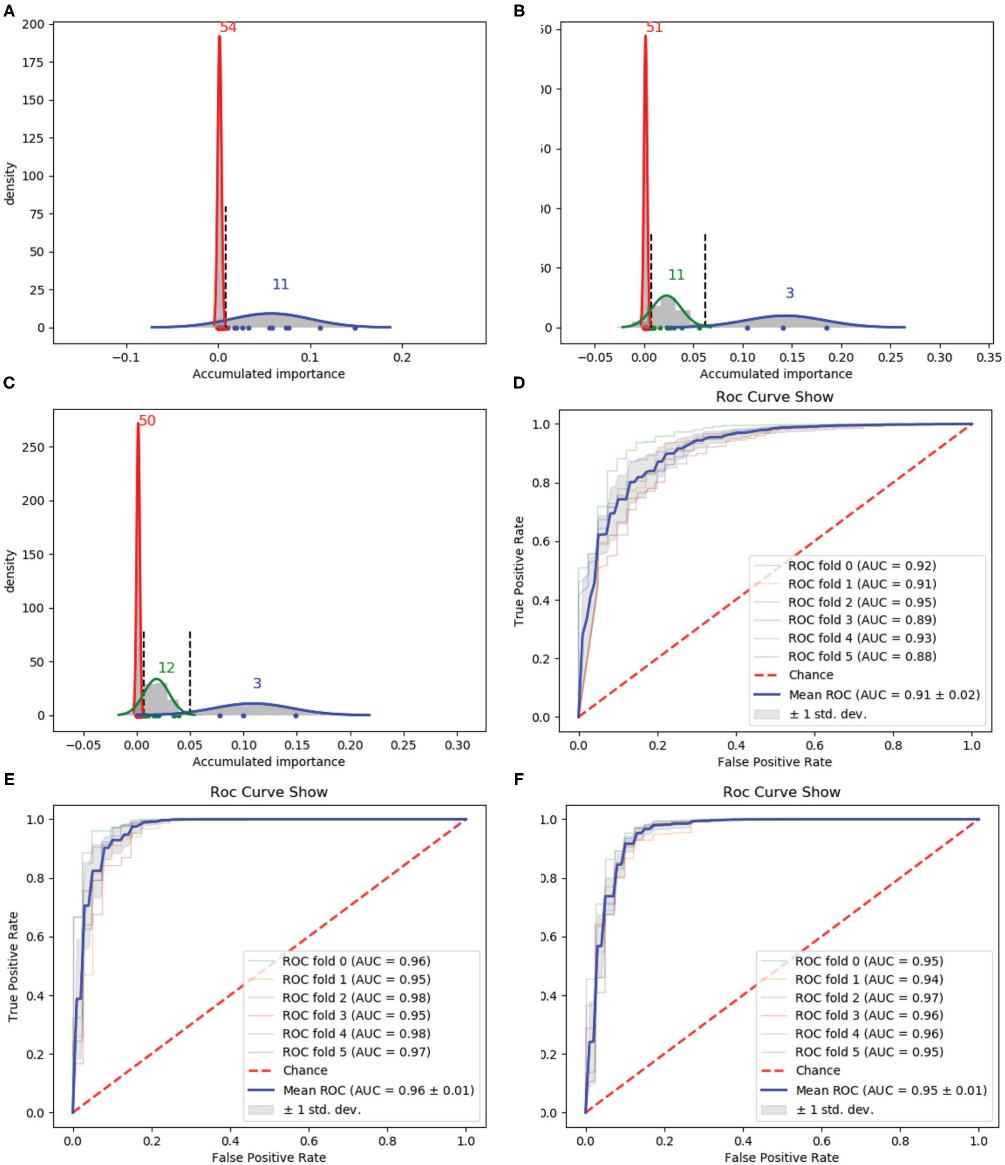
Qualitative results on PAAC. **(A–C)** Gaussian distribution of the accumulated variable importance corresponding to three-time random sample splits, respectively. **(D–F)** Refer to the ROC curves and AUC values of the selected two variables, PAAC and the selected eight variables, respectively.

Then, we used PAAC as the original feature for variable selection. Following the same way as 188D, we also performed 1 × 10^5^ rounds to stabilize the results and repeated the procedure three times on three groups of randomly selected training samples. Gaussian distributions of the GMM instances fitted by the accumulated variable importance are shown in Figures 3A–C, respectively.

Figure 3A illustrates two Gaussian mixture components from right to left, each component of which contains 11 and 54 variables with the variable importance in a descending order. Figure 3B presents three, 11 and 51 variables in the three Gaussian distributions from right to left with the variable importance in a descending order. Meanwhile, three, 12 and 50 variables are included in three Gaussian components from right to left, respectively, as shown in Figure 3C.

We first select the variables in subset *Q*, which is the interaction of variables belonging to the Gaussian component with the highest mean score in Figure 3, variables belonging to the Gaussian component with the highest mean score in Figure 3B and variables belonging to the Gaussian component with the highest mean score in Figure 3C, namely, *Q* = (*G*_11_) ∩ (*G*_21_) ∩ (*G*_31_). Here, *i* is the index of the three times and *G*_*ij*_ denotes the Gaussian distribution which has the *j*th highest mean score of variable importance in the *i*th time. *Q* consists of only two variables. The corresponding ROC curves and AUC values can be seen in Figure 3D.

If we expand the scope by including two green Gaussian components illustrated in Figures 3B,C, namely, we now select variables in subset *Q*′, where *Q*′ is defined as $Q^{\prime }=\left(G_{11}\right)\cap \left(G_{21}\cup G_{22}\right)\cap \left(G_{31}\cup G_{32}\right),$ eight components will be selected. The corresponding ROC curves and AUC values can be seen in Figure 3F. Besides, the ROC curves and AUC values of PAAC are shown in Figure 3E.

Again, similar to what we have done on 188D, starting from the set *A* consisting of the only variable with the highest importance score, we progressively add to *A* new elements in *Q* which is made up of originally selected two variables with their importance in a descending order and present quantitative results in Table 2. Then, detailed results of the selected two variables together with the eight variables and PAAC are listed in Table 2.

**Table 2:** Quantitative results on PAAC.

**Feature**	**Confusion matrix**			**Class**	**TP rate**	**FP rate**	**Precision**	**Recall**	**F1-measure**
(10)^*T*^	Classified as − >	a	b	a: Positive	0.246	0.034	0.267	0.246	0.256
	a	60	184	b: Positive	0.966	0.754	0.962	0.966	0.964
	b	165	4,630	Weighted average	0.931	0.719	0.929	0.928	0.930
(10, 1)^*T*^	Classified as − >	a	b	a: Positive	0.471	0.012	0.669	0.471	0.553
	a	115	129	b: Positive	0.988	0.529	0.973	0.988	0.980
	b	57	4,738	Weighted average	0.963	0.504	0.958	0.963	0.959
(10, 1, 30,	Classified as − >	a	b	a: Positive	0.652	0.001	0.958	0.652	0.776
29, 22, 9,	a	159	85	b: Positive	0.999	0.348	0.983	0.999	0.991
12, 13)^*T*^	b	7	4,788	Weighted average	0.982	0.331	0.982	0.982	0.981
PAAC	Classified as − >	a	b	a: Positive	0.643	0.000	0.994	0.643	0.781
	a	157	87	b: Positive	1.000	0.357	0.982	1.000	0.991
	b	1	4,794	Weighted average	0.983	0.340	0.983	0.983	0.981

**Figure 4:**
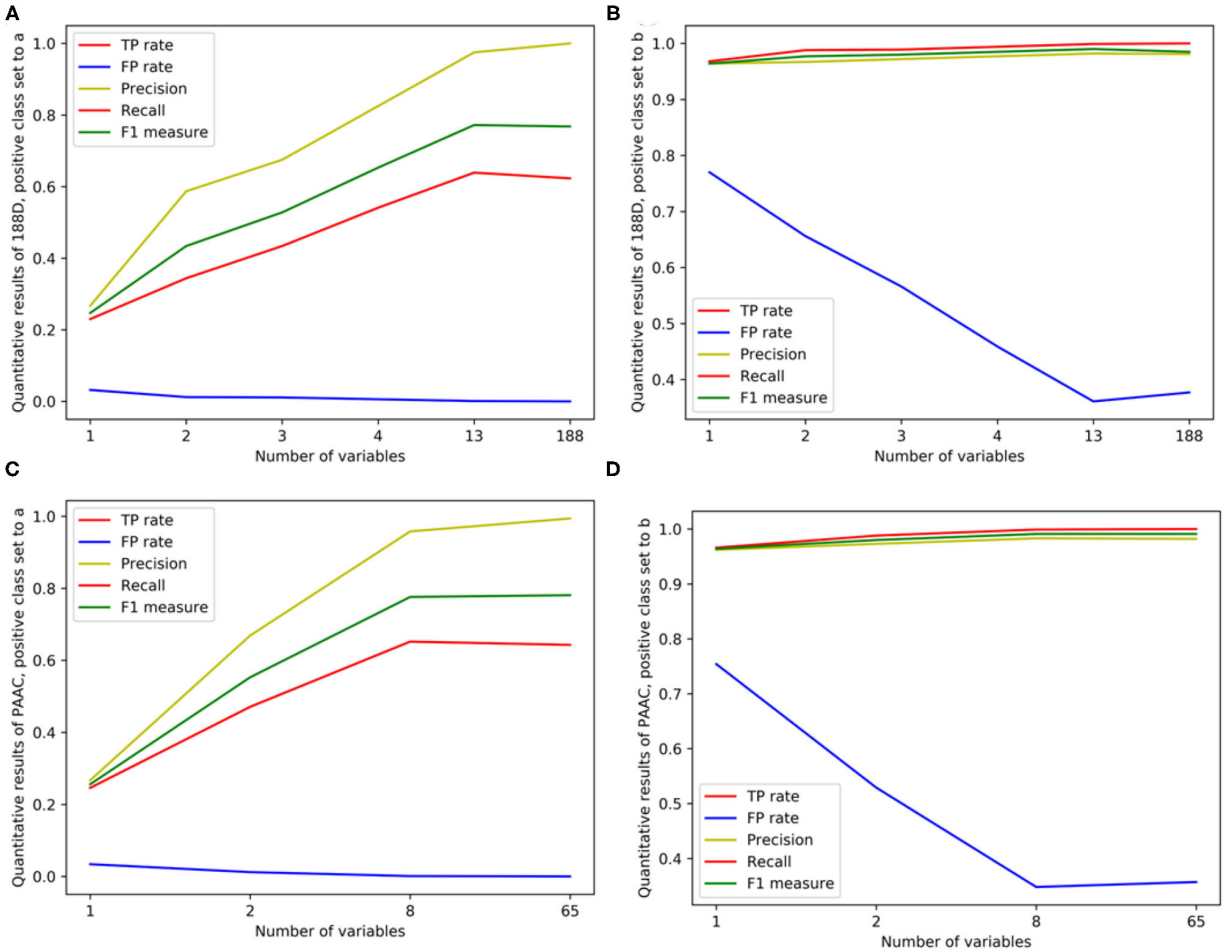
Line charts of quantitative results on 188D and PAAC, with the x axis representing the number of selected variables. **(A,B)** Quantitative results on 188D with the positive class set to *a* and *b*, respectively. **(C,D)** Quantitative results on PAAC with the positive class set to *a* and *b*, respectively.

In Table 2, the confusion matrix, true positive (TP) rate, false positive (FP) rate, precision, recall, and F1 measure are also presented for the results corresponding to the compound of selected variables. The two classes representing PPR positive samples (labeled *a*) and negative ones (labeled *b*) are separately considered as positive classes when we calculate these quantitative results. As more and more variables are added to *Q*, the frequency of misclassifying samples labeled *b* to *a* decreased and vice versa; while the TP rate using both class *a* and class *b* as positive class improves, so do the precision, recall and F1 measure. When setting the positive class to *b* and the error rate of misclassifying samples of label *a* to *b*, the FP rate also follows a similar trend of the result on 188D. These changes of quantitative results regarded as the function of the variable number are plotted in Figures 4C,D, respectively. It can be also seen that the eight variables keep a comparable result with PAAC.

### 3.3. Results on Low-Redundant Training Data From 20-Fold Sample Split

Actually, we don't know whether the dataset representing plant PPR (Qu et al., 2019) is non-redundant or not. And the case might be that redundancy exists among 9590 PPR negative sequences. However, some statistical strategies can be employed to reduce data redundancy as much as possible. Here, we used all the 243 positive samples of the training set, and divided the 4,795 negative samples of the training set equally into 20 sets. We made this 20-fold sample split to make a balance between the number of PPR positive proteins and that of negative ones. This kind of strategy may help to reduce redundancy among PPR negative samples. Again, this procedure was repeated two times, each of which corresponded to a randomly selected original group of training samples.

**Figure 5:**
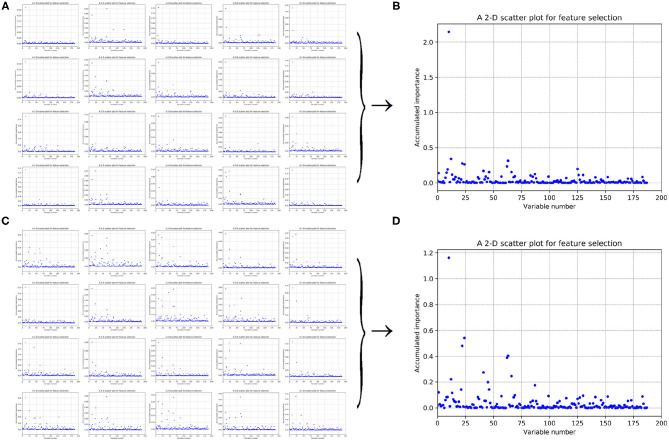
Scatter plots derived from feature 188D, each of which keeps x and y axis representing variable and its importance score, respectively. **(A,C)** Correspond to 20-fold sample split from twice random generations of the training samples. **(B,D)** Refer to the accumulated results of the 20-fold scatter plots in **(A,C)**, respectively.

**Figure 6:**
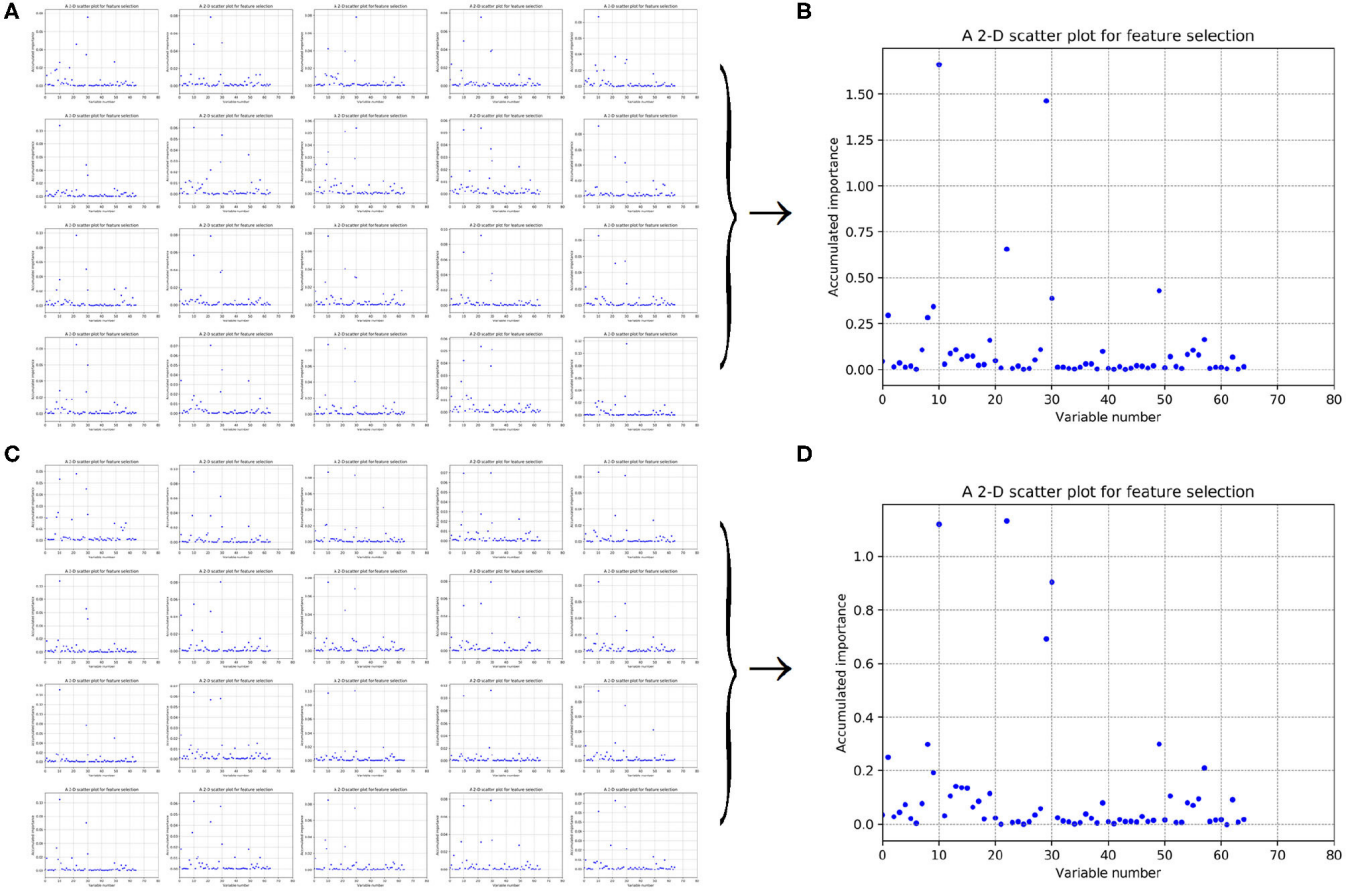
Scatter plots derived from feature PAAC, each of which keeps x and y axis representing variable and its importance score, respectively. **(A,C)** Correspond to 20-fold sample split from twice random generations of the training samples. **(B,D)** Refer to the accumulated results of the 20-fold scatter plots in **(A,C)**, respectively.

As to each fold of negative samples and the 243 positive ones, we followed the resampling, training, and scoring step in section 2.2 and made 1 × 10^4^ rounds of the iteration. Correspondingly, a scatter plot was obtained, with its x and y coordinate representing each variable and its importance score, respectively. The experimental results on 188D and PAAC are shown in Figures 5, 6, respectively.

Figures 5A,C refer to twice random generations of the training set. Twenty scatter plots corresponding to 20-fold negative samples are listed in each sub-figure. Besides, two scatter plots which record the accumulated scores of variable importance are listed in Figures 5B,D, respectively. It can be seen that variable 10 in 188D is obviously more important than the other variables.

As to Figures 6A,C, it refers to twice random generations of the training set. Twenty scatter plots corresponding to 20-fold negative samples can be seen in each sub-figure. Moreover, two scatter plots which correspond to the accumulated scores of variable importance are shown in Figures 6B,D, respectively. It can be also seen that variable 10 in PAAC is important.

**Figure 7:**
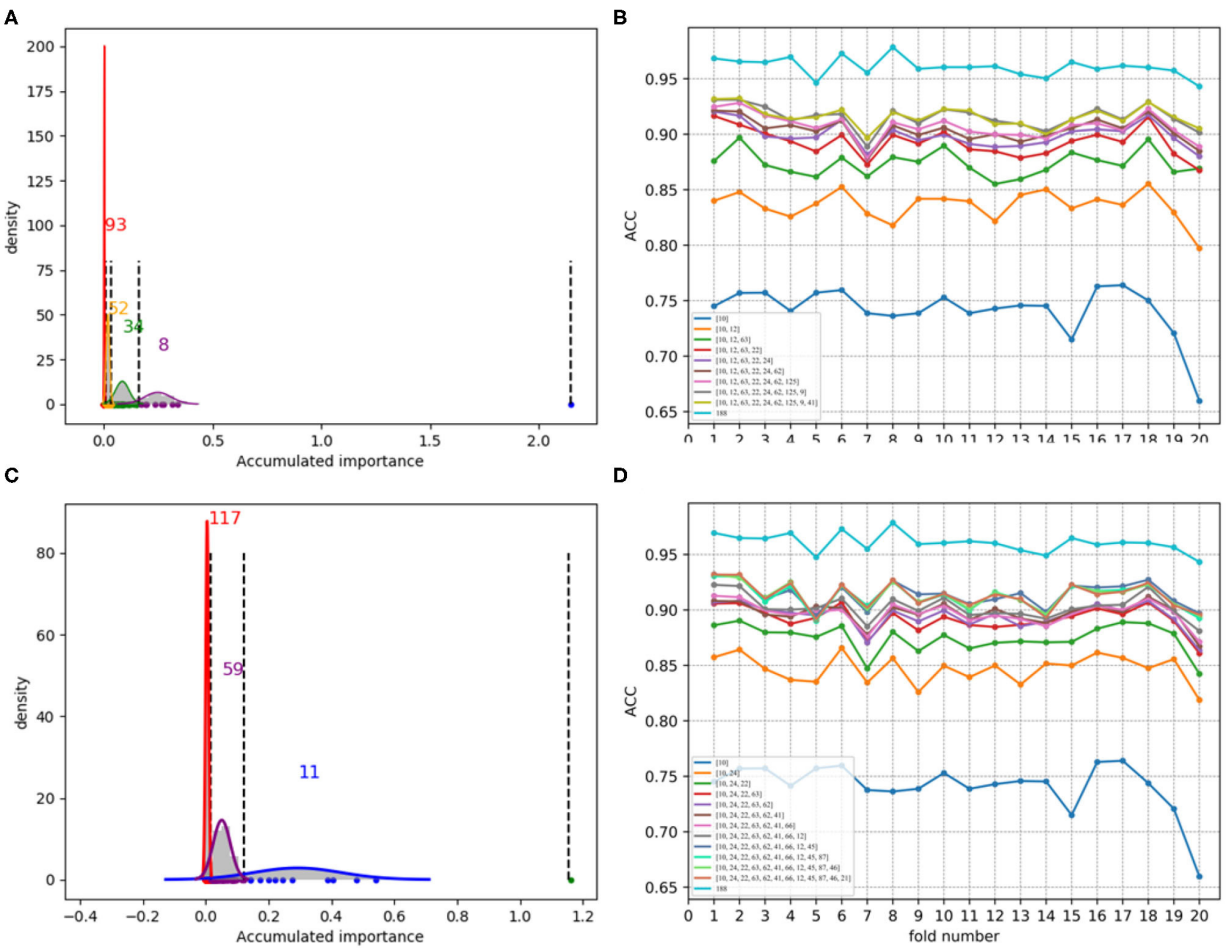
Qualitative results on low-redundant dataset using 188D. **(A,C)** Gaussian distributions of the accumulated variable importance corresponding to twice random sample splits, respectively. **(B,D)** Refer to ACC line charts of different dimensions derived from feature 188D, with important variables incrementally added one by one according to the accumulated scores in a descending order.

**Figure 8:**
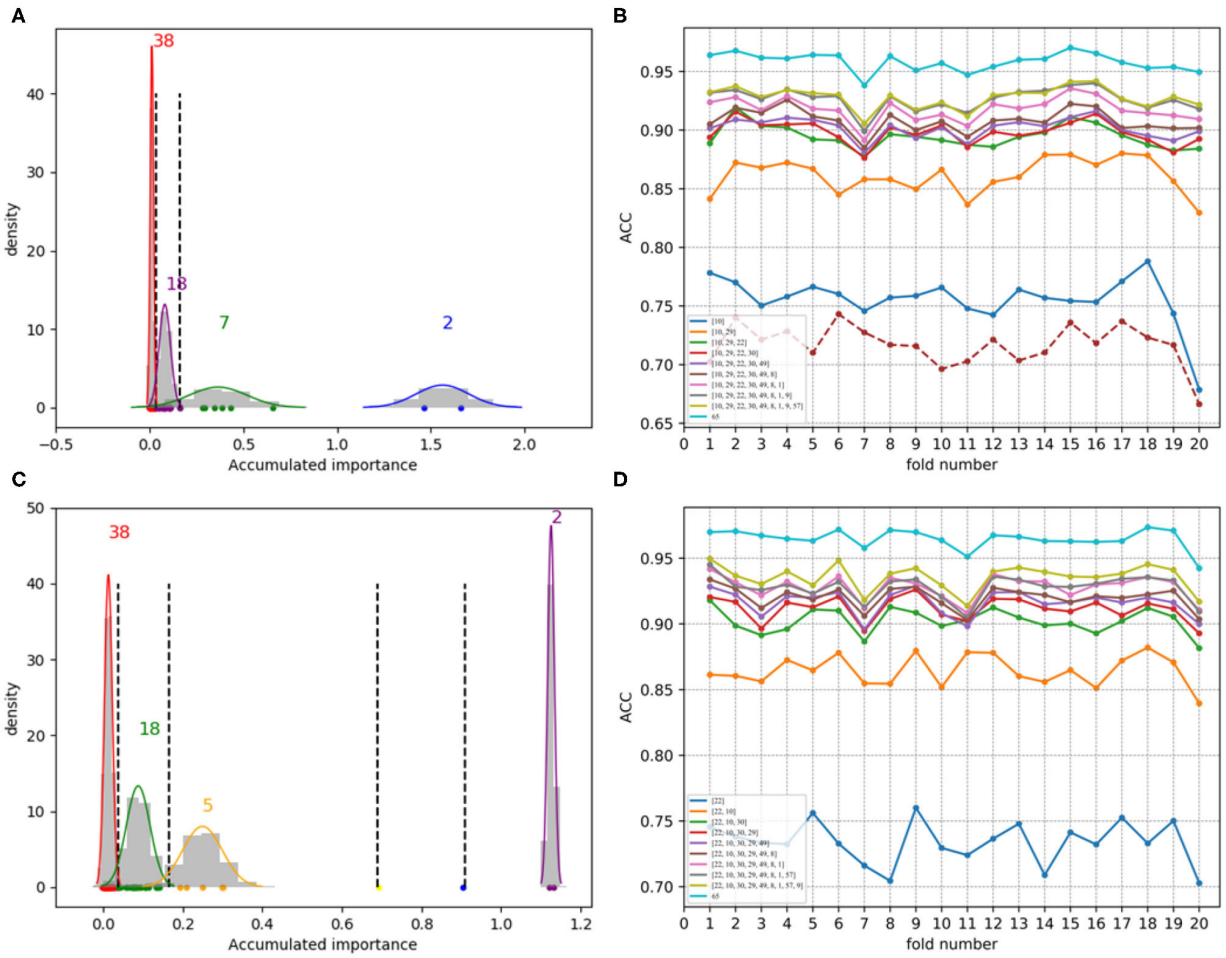
Qualitative results on low-redundant dataset using PAAC. **(A,C)** Gaussian distributions of the accumulated variable importance corresponding to twice random sample splits, respectively. **(B,D)** Refer to ACC line charts of different dimensions derived from feature PAAC, with important variables incrementally added one by one according to the accumulated scores in a descending order.

As having been stated in section 2.3, after making automatic variable selection on accumulated scores of variable importance shown in Figures 5B,D, 6B,D, Gaussian distributions of the accumulated variable importance corresponding to the twice random generations of the training set are obtained and illustrated in Figures 7, 8, respectively.

Figures 7A,C, which are associated with the scatter plots in Figures 5B,D, show Gaussian distributions of the accumulated variable importance using feature 188D. As to Figures 8A,C, they correspond to the scatter plots in Figures 6B,D and represent Gaussian distributions of the accumulated variable importance using feature PAAC. In practice, we regard variables that belongs to the first two Gaussian mixture components (or outliers) with higher scores of variable importance as important variables.

### 3.4. Results on Low-Redundant Testing Data From 20-Fold Sample Split

Now that important variables were selected according to Gaussian distributions derived from each training set, we moved on to the testing set. In order to get low-redundant data, we divided negative samples of the testing set equally into 20 sets, each of which kept a comparable number as that of the positive samples. Each set of the negative samples and all the positive ones together formed a fold on the testing set.

In each fold, we followed the resampling, training, and scoring step stated in section 2.2 and made 1 × 10^4^ rounds of the iteration. Therefore, a random forest containing 1 × 10^4^ CARTs was built. Then, the left samples on the testing set were used to calculate an ACC value, as having been expressed in Equation (9). Each fold corresponded to an ACC value. As a result, we could get a line chart including 20 ACC values in a specific dimensional space.

Since important variables have been selected in each random generation of the training set using either feature 188D or feature PAAC, important variables can be sorted according to their accumulated scores in a descending order. Following this order, variables can be incrementally added so that different dimensional spaces composed of important variables are established.

Figures 7B,D, which are separately associated with Figures 7A,C, show ACC line charts of different dimensions derived from feature 188D. The line chart keeping the lowest ACC values corresponds to variable 10 in 188D; while, the one keeping the highest ACC values represents feature 188D. It can be seen that ACC values may increase with the addition of feature dimension.

As to Figures 8B,D, which are separately associated with Figures 8A,C, ACC line charts of different dimensions derived from feature PAAC are also listed in turn. The variable with the highest score of variable importance keeps the lowest ACC values; while, the one keeping the highest ACC values corresponds to feature PAAC. Also, it can be discovered that ACC values may increase with the addition of feature dimension.

From the ACC lines charts of different dimensions shown in Figures 7, 8, we see that ACC values increase with the growth of feature dimensions. Anyway, even though all the important variables are used, the ACC value is still slightly less better than the ACC value using feature 188D or feature PAAC. We wonder at which dimension the incrementally added variables can obtain almost same ACC values as feature 188D or feature PAAC does.

**Figure 9:**
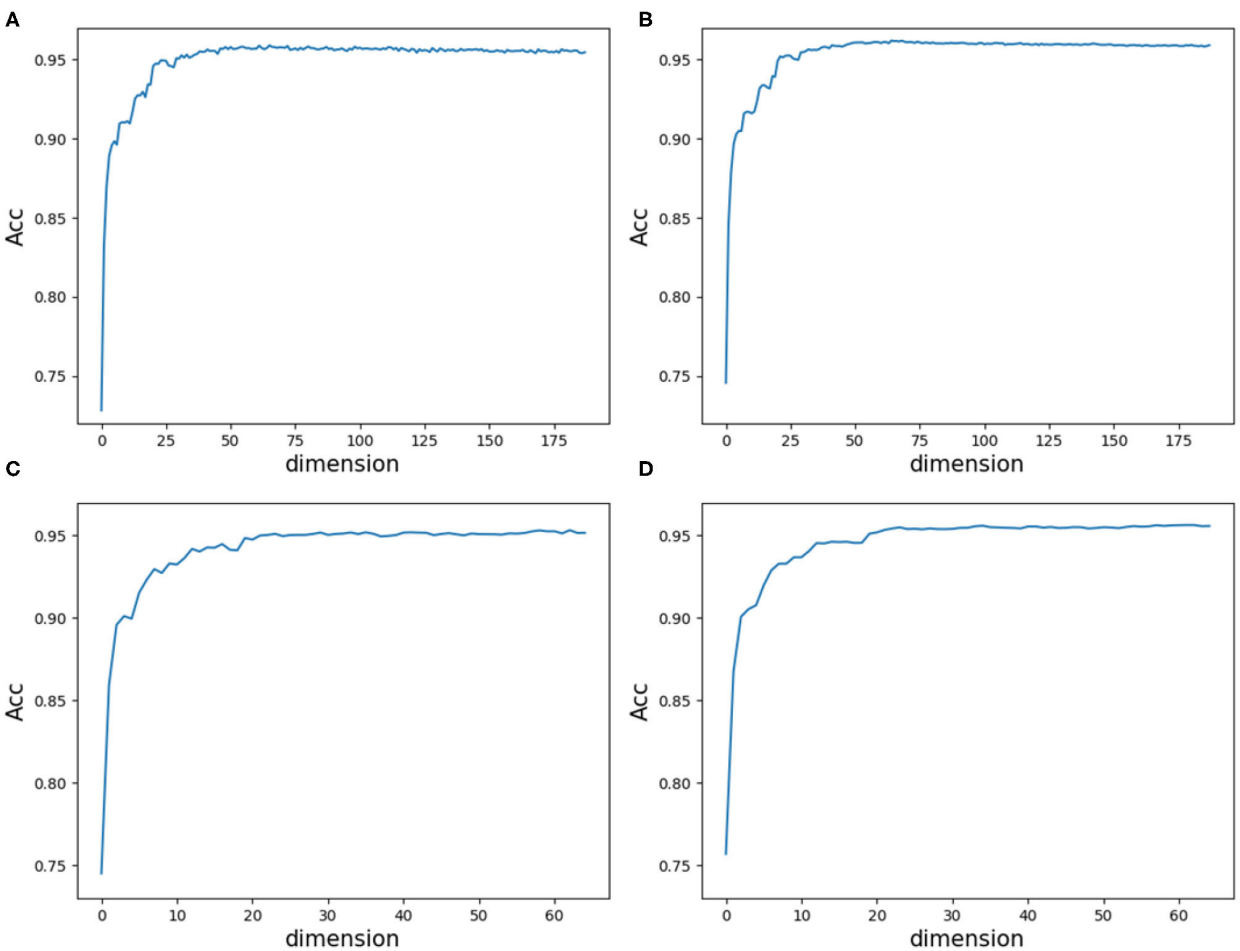
Line charts of the average ACC values with increasing feature dimensions following the order of the variable importance. **(A)** Is the line chart derived from the average of 20-fold cross validation using 188D. **(B)** Is the line chart derived from the average of 1,000 times equal resampling on negative samples from a testing set compared with the number of positive samples on the testing set using 188D. **(C)** Is the line chart derived from the average of 20-fold cross validation using PAAC. **(D)** Is the line chart derived from the average of 1,000 times equal resampling on negative samples from a testing set compared with the number of positive samples on the testing set using PAAC.

As a result, we followed the order of the variable importance, made 1 × 10^4^ rounds of the iteration by repeating the resampling, training and scoring step to establish a random forest with 1 × 10^4^ CARTs, and obtained line charts of the average ACC values in different dimensions. Figure 9 illustrates the experimental results in detail. It can be indicated that the first 25 variables may achieve almost the same ACC values as feature 188D or feature PAAC does.

By making a comparison between Figures 8B,D, we found the ACC line charts of the most important variable in these two subfigures are of different ACC values. In fact, the most important variable shown in Figure 8B is variable 10 in PAAC; while, it is variable 22 in Figure 8D. Thus, we inferred variable 10 is more important. After attaching the ACC line chart of variable 22 using dotted line, we found the line chart of variable 10 is above that of variable 22. That is to say, variable 10 should be the most important variable in PAAC despite the instability of selected variables using PAAC (see Figures 6B,D).

**Figure 10:**
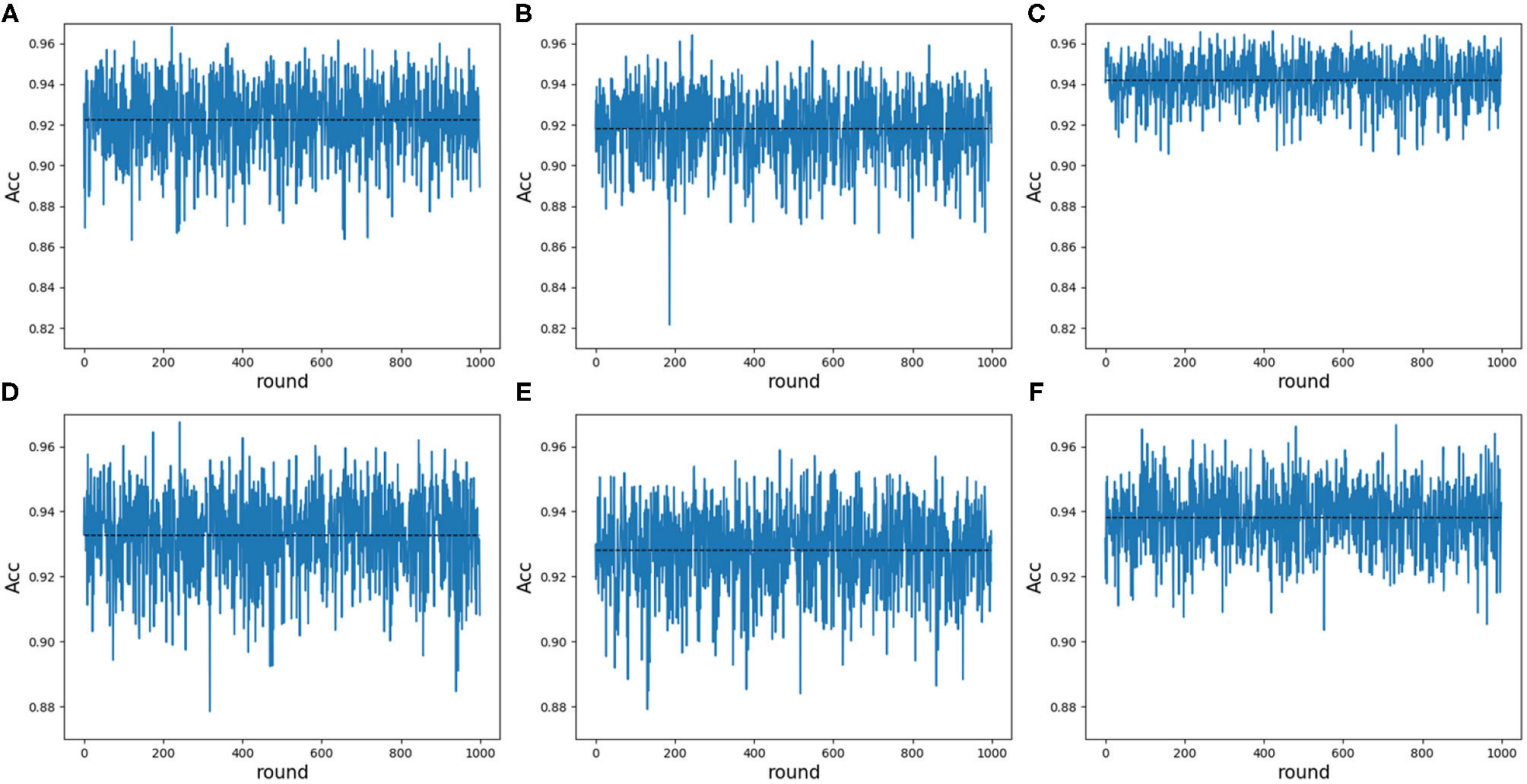
Line charts of ACC values in 25 dimensions including and excluding variable 10 on 188D and PAAC. The black dotted line refers to the average ACC values of 1,000 rounds. **(A)** Shows the ACC values of any 25 dimensional feature randomly extracted from 188D in each round of 1,000 times negative sample extraction. **(B)** Illustrates the ACC values of any 25 dimensional feature randomly extracted from 188D after excluding variable 10 in each round of 1,000 times negative sample extraction. **(C)** Shows the ACC values of any 25 dimensional feature including variable 10 that is randomly extracted from 188D in each round of 1,000 times negative sample extraction. **(D)** Shows the ACC values of any 25 dimensional feature randomly extracted from PAAC in each round of 1,000 times negative sample extraction. **(E)** Illustrates the ACC values of any 25 dimensional feature randomly extracted from PAAC after excluding variable 10 in each round of 1,000 times negative sample extraction. **(F)** Shows the ACC values of any 25 dimensional feature including variable 10 that is randomly extracted from PAAC in each round of 1,000 times negative sample extraction.

Note that 188D and PAAC keep the same variable 10. In order to validate whether variable 10 plays a part in predicting PPR proteins, we randomly extracted negative samples with the number equal to that of positive samples on a testing set and made 1 × 10^4^ rounds of resampling, training and scoring step to form a random forest. The left samples were used to calculate ACC values. The random extraction was repeated 1,000 times. Figure 10 shows the experimental results in detail. It can be seen that any 25 dimensional feature excluding variable 10 shown in Figures 10B,E has a lower average ACC value than that of any 25 dimensional feature (see Figures 10A,D). As to any 25 dimensional feature including variable 10, it keeps a higher average ACC value (see Figures 10C,F) than that of any 25 dimensional feature. Thus, it indicated that variable 10 really plays an important role in predicting PPR proteins.

### 3.5. Results on Non-redundant Data

In order to show the effectiveness of our method, redundancy has to be removed from the dataset representing plant PPR (Qu et al., 2019). A redundancy removing tool namely Cd-hit (Li and Adam, 2006) is used at 25% cutoff, which means no two protein sequences have similarity more than 25%. This redundancy removing procedure was made on 487 PPR positive protein sequences and 9,590 negative ones, respectively. Correspondingly, 170 PPR positive proteins and 9,293 negative ones were left, and they composed the non-redundant data. As shown in Figure 1, a balanced sample split was made. That is, 85 PPR positive proteins together with 4,646 negative ones were randomly selected as the training set. The left proteins composed the testing set. Again, this procedure was repeated two times, each of which corresponded to a random selection of training samples.

**Figure 11:**
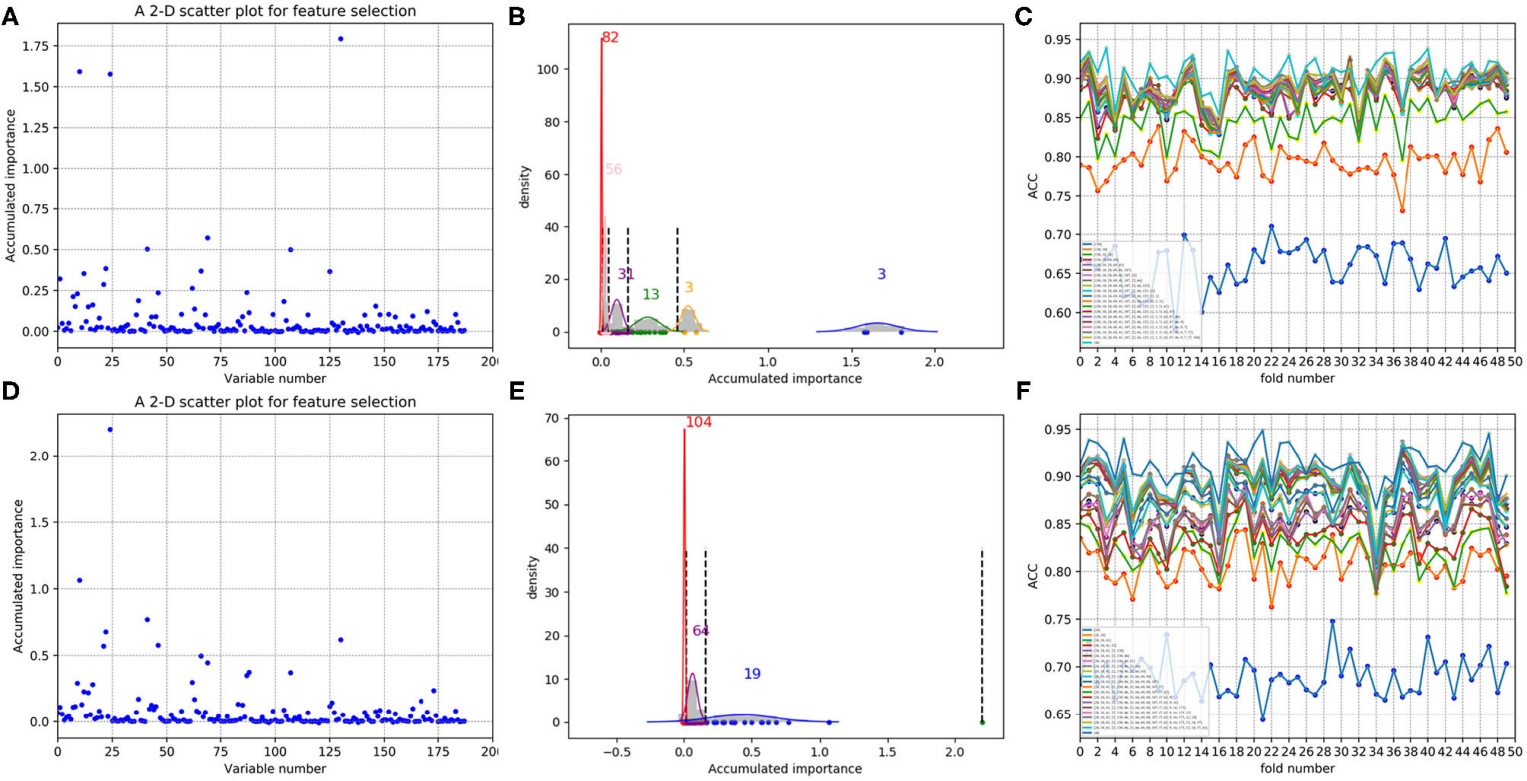
Qualitative results on non-redundant dataset using 188D. **(A,D)** Refer to the accumulated results of the 50-fold scatter plots, each of which corresponds to one time of random sample selection for training. **(B,E)** Gaussian distributions of the accumulated variable importance corresponding to twice random sample splits, respectively. **(C,F)** ACC line charts of different dimensions derived from feature 188D, with important variables incrementally added one by one according to the accumulated scores in a descending order.

**Figure 12:**
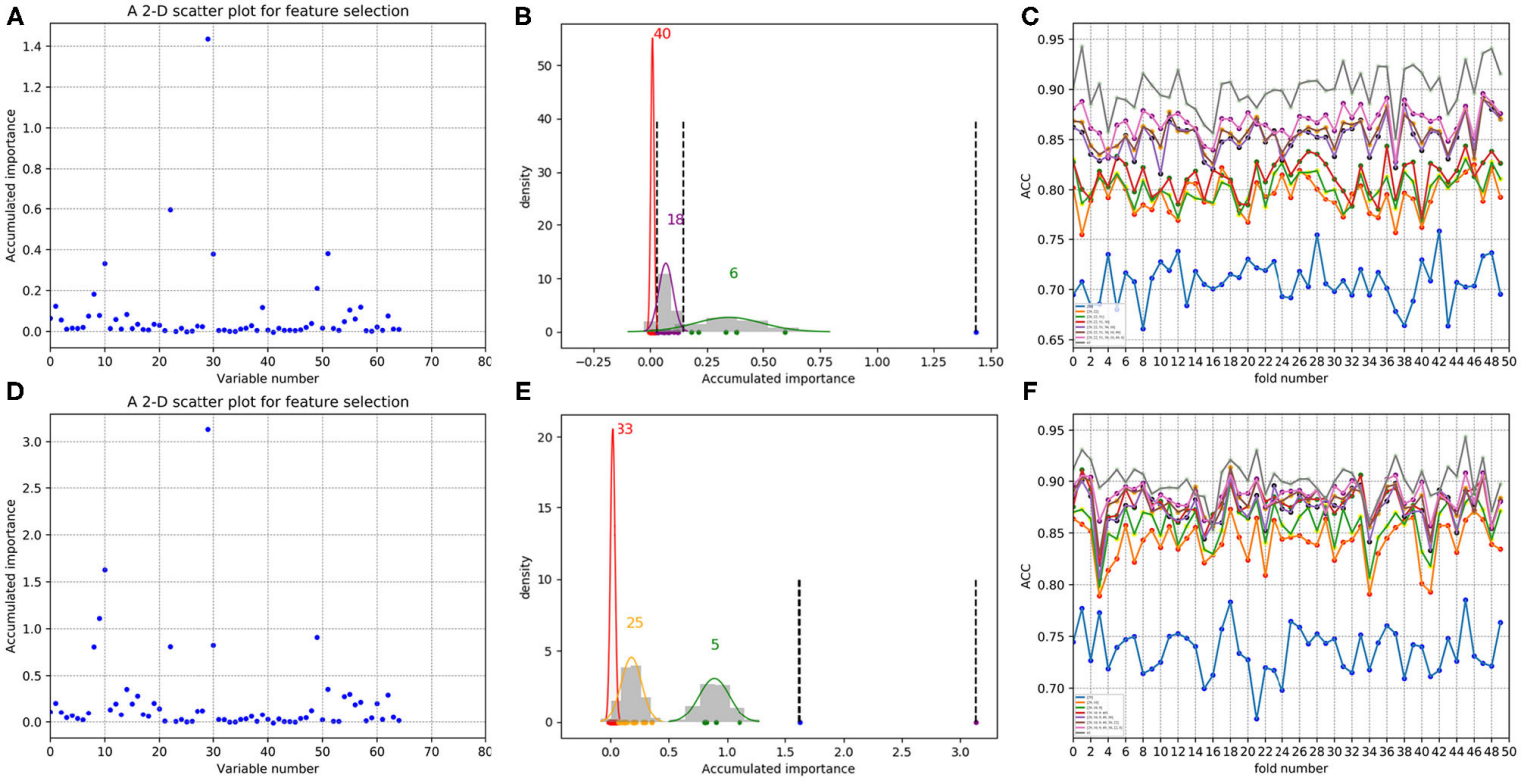
Qualitative results on non-redundant dataset using PAAC. **(A,D)** Refer to the accumulated results of the 50-fold scatter plots, each of which corresponds to one time of random sample selection for training. **(B,E)** Gaussian distributions of the accumulated variable importance corresponding to twice random sample splits, respectively. **(C,F)** Illustrate ACC line charts of different dimensions derived from feature PAAC, with important variables incrementally added one by one according to the accumulated scores in a descending order.

In each time of random sample selection for training, we divided the 4,646 negative proteins of the training set equally into 50 sets in order to make a balance between the positive and negative samples. As to each fold of negative samples and the 85 positive ones, we followed the resampling, training and scoring step in section 2.2 and made 1 × 10^4^ rounds of the iteration. After traversing all the 50-folds, a scatter plot recording the accumulated scores of variable importance was obtained, with its x and y coordinate representing each variable and its importance score, respectively. The experimental results on 188D and PAAC are shown in Figures 11, 12, respectively. Figures 11A,D show the scatter plots derived from feature 188D, each of which corresponds to one time of random sample selection for training. Gaussian distributions of the accumulated variable importance corresponding to the twice random selection of the training set are illustrated in Figures 11B,E, respectively. Accordingly, experimental results using feature PAAC are shown in Figures 12A,B,D,E, respectively.

As to each testing set, 4,647 negative samples were equally divided into 50 sets, each of which kept a comparable number as that of the 85 positive samples. Each set of the negative samples and all the positive samples formed a fold. Thus, we obtained 50-folds. In each fold, we followed the automatic variable selection step shown in Figure 1, which has been clearly stated in section 3.4. A line chart including 50 ACC values could be obtained in a specific dimensional space deriving from important variables incrementally added according to their accumulated scores in a descending order. Therefore, Figures 11C,F show ACC line charts of different dimensions derived from 188D. Correspondingly, experimental results using feature PAAC are illustrated in Figures 12C,F, respectively.

**Figure 13:**
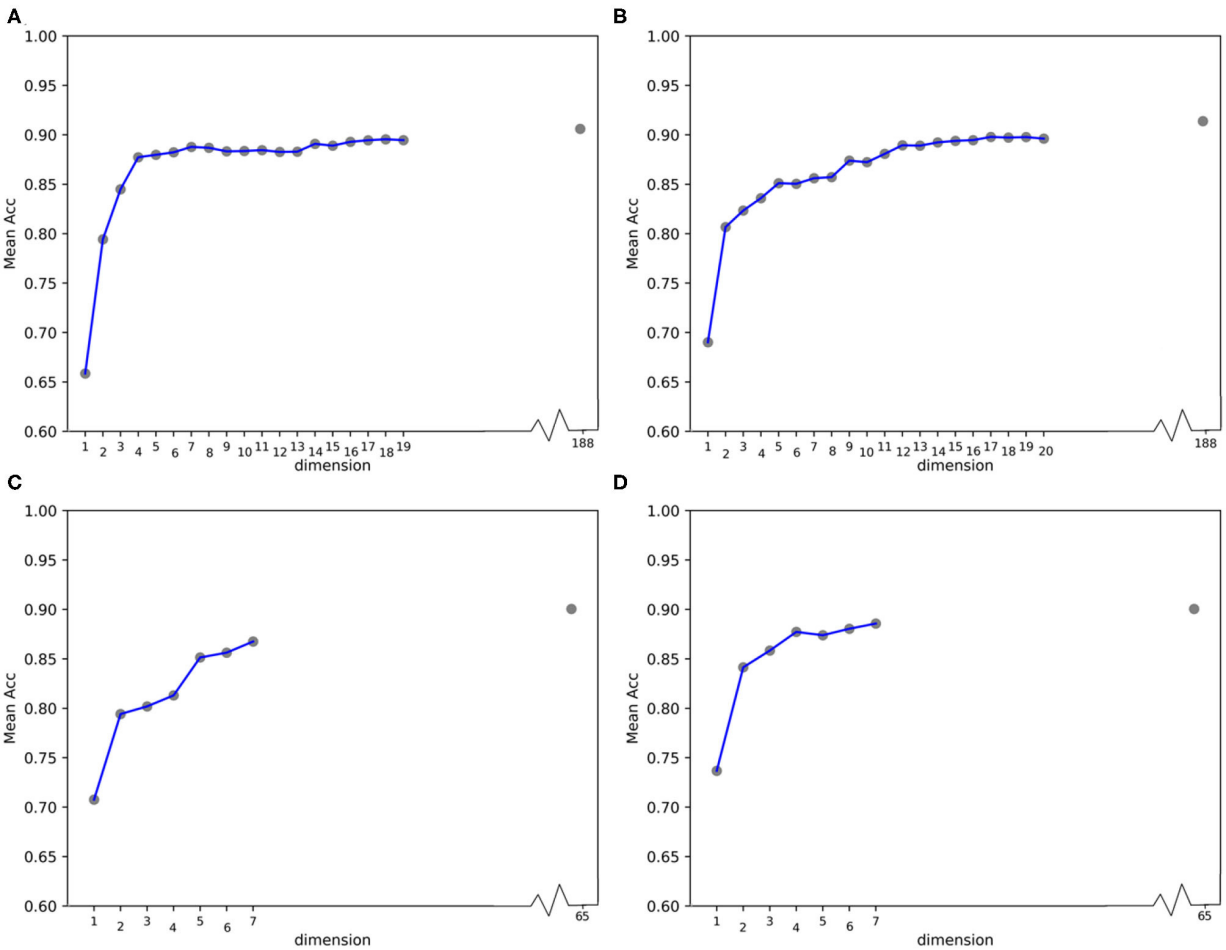
Line charts of the average ACC values with increasing feature dimensions following the order of the variable importance on non-redundant dataset. **(A,B)** Are the line charts derived from the average of 50-fold cross validation using 188D, which correspond to twice random sample splits. **(C,D)** Are the line charts derived from the average of 50-fold cross validation using feature PAAC, which correspond to twice random sample splits.

Furthermore, we calculated the average ACC values of the 50 folds derived from feature 188D and PAAC in different dimensions, as shown in Figure 13. Figures 13A,B correspond to the experimental results of the twice random selection of the training set using feature 188D. As to Figures 13C,D, it refers to the experimental results of the twice random selection of the training set using feature PAAC. It can be discovered that mean ACC values may increase when enlarging feature dimension. The selected variables regarded to be important always obtain ACC values comparable to those of feature 188D or PAAC, which indicates the effectiveness of the selected variables. After making a comparison between Figure 9 and Figure 13, we find that our variable selection method still works on non-redundant data despite the existence of 5% point loss on the average ACC values from feature 188D and feature PAAC.

## 4. Discussions

According to the experimental results, we make some discussions as follows. Firstly, it needs to be considered whether the classification accuracy will come down when non-redundant data is used. It is observed that methods presented by Qu et al. (2019) and the 20-fold sample split in this paper have used all the PPR positive proteins for training, which means there are only negative samples in a testing set. In that case, ACC is equivalent to TP rate, for PPR negative proteins are labeled with positive class (see Equation 9). Table 1 shows the classification results on a testing set containing 244 PPR positive proteins and 4,795 negative ones. Correspondingly, the training set which includes 243 PPR positive proteins and 4,795 negative ones is considered to be most redundant. As listed in Table 1, the ACC values (i.e., the TP rate when class b is labeled to be positive) using feature 188D and only variable 10 are 1.000 and 0.968. As to the low-redundant testing data from 20-fold sample split, only 243 PPR positive proteins and no more than 240 negative samples are used for training, which shows more lest redundancy (only 9.6% of the former training sample size). It can be discovered in Figure 8 that the ACC values are commonly above 0.95 when the dimension of selected variables is bigger than 25. That means the classification accuracy will come down a little but not drastically when low-redundant data is used. As to non-redundant data, experimental results shown in Figure 13 exhibit that the mean ACC values are approximate to 0.9 with the feature dimension increasing, which demonstrates the effectiveness of our method.

Secondly, we want to discuss whether variable 10 is effective for identifying PPR proteins and whether all the variables in feature 188D or feature PAAC are needed. The experimental results in Figure 10 indicate the importance of using variable 10 for classification. The selected four variables (10, 12, 130, 1)^*T*^ shown in Table 1 correspond to the content of methionine, proline, cysteine, and the conversion frequency of amino acid surface tension, respectively. Meanwhile, the selected two variables (10, 1)^*T*^ shown in Table 2 refer to the occurrence frequencies of cysteine and methionine in PPR proteins. Thus, it can be inferred that the content of methionine in proteins plays an important role in predicting PPR proteins. Besides, it can be inferred from Figure 9 that 25 dimensional features instead of feature 188D or feature PAAC may also work. Despite the phenomenon that variable 10 is not selected as the most important variable (see Figures 11, 12), it may because of redundancy removal that makes the number of PPR positive proteins down from 487 to 170.

Thirdly, whether classification methods should be used for identifying proteins with specific functions needs to be discussed. As shown in Tables 1, 2, the quantitative results regarding PPR negative proteins as samples with positive class labels (i.e., *b* to positive) are often better than those regarding PPR positive proteins as samples with positive class labels (i.e., *a* to positive). After observing the confusion matrix, it is found that samples regarded as PPR positive proteins are wrongly classified in a high rate. In fact, these PPR positive proteins are derived from UniPort by searching the keyword “pentatricopeptide repeat” (Qu et al., 2019), it is possible that the proteins searched out emerge as false positives due to lack of biological validation. In that case, it may be better if we use clustering methods for partitioning proteins regarded as positive in advance.

## 5. Conclusion

PPR proteins play a vital role in plant growth and development. In this study, we proposed a framework of variable selection for predicting PPR proteins. A random sample split was made for obtaining a training and a testing set in balance. An iteration referred to resampling, training, and scoring step was implemented to stabilize the results of variable selection. Then, important variables were automatically selected by employing GMM with BIC. Qualitative and quantitative results demonstrated that the content of methionine may play an important role in predicting PPR proteins. Besides, important variables other than the extracted feature are applicable to prediction of PRR proteins. In future work, clustering methods will be considered in advance for getting better identifying results.

## Data Availability Statement

The original contributions presented in the study are included in the article/supplementary files, further inquiries can be directed to the corresponding author/s.

## Author Contributions

GW conceived the general project and supervised it. XZ and GW initiated the idea, conceived the whole process, and finalized the paper. HW and HL were the principal developers and made the supplementary experiments. YW helped to modify the manuscript. All authors read and approved the final manuscript.

## Conflict of Interest

The authors declare that the research was conducted in the absence of any commercial or financial relationships that could be construed as a potential conflict of interest.

## References

[B1] Chen G. Zou Y. Hu J. Ding Y. (2018). Genome-wide analysis of the rice PPR gene family and their expression profiles under different stress treatments. BMC Genomics 19:720. 10.1186/s12864-018-5088-9 30285603 PMC6167770

[B2] Chou K. (2001). Prediction of protein cellular attributes using pseudo-amino acid composition. Proteins 43, 246–255. 10.1002/prot.1035 11288174

[B3] Chou K. (2005). Using amphiphilic pseudo amino acid composition to predict enzyme subfamily classes. Bioinformatics 21, 10–19. 10.1093/bioinformatics/bth466 15308540

[B4] Li R. Perneczky R. Yakushev I. Förster S. Kurz A. Drzezga A.. (2015). Gaussian mixture models and model selection for [18f] fluorodeoxyglucose positron emission tomography classification in Alzheimer's disease. PLoS ONE 10:e0122731. 10.1371/journal.pone.0122731 25919662 PMC4412726

[B5] Li W. Adam G. (2006). CD-hit: a fast program for clustering and comparing large sets of protein or nucleotide sequences. Bioinformatics 22, 1658–1659. 10.1093/bioinformatics/btl158 16731699

[B6] Li Y. Niu M. Zou Q. (2019). ELM-MHC: An improved MHC identification method with extreme learning machine algorithm. J. Proteome Res. 18, 1392–1401. 10.1021/acs.jproteome.9b00012 30698979

[B7] Lv Z. Jin S. Ding H. Zou Q. (2019). A random forest sub-golgi protein classifier optimized via dipeptide and amino acid composition features. Front. Bioeng. Biotechnol. 7:215. 10.3389/fbioe.2019.00215 31552241 PMC6737778

[B8] Nan Y. Chai K. Lee W. Chieu H. (2012). Optimizing f-measure: a tale of two approaches, in the 29th International Conference on Machine Learning (ICML2012), eds Langford J. Pineau J. (Edinburg: Omni Press), 289–296.

[B9] Qu K. Wei L. Yu J. Wang C. (2019). Identifying plant pentatricopeptide repeat coding gene/protein using mixed feature extraction methods. Front. Plant Sci. 9:1961. 10.3389/fpls.2018.01961 30687359 PMC6335366

[B10] Rojas M. Ruwe H. Miranda R. G. Zoschke R. Hase N. Schmitz-Linneweber C.. (2018). Unexpected functional versatility of the pentatricopeptide repeat proteins PGR3, PPR5 and PPR10. Nucleic Acids Res. 46, 10448–10459. 10.1093/nar/gky737 30125002 PMC6212717

[B11] Ru X. Li L. Zou Q. (2019). Incorporating distance-based top-n-gram and random forest to identify electron transport proteins. J. Proteome Res. 18, 2931–2939. 10.1021/acs.jproteome.9b00250 31136183

[B12] Ruida G. Zeng F. Zhan Y. Li S. Geng H. Zhang G.. (2013). Variation analysis of traits of seeds on interspecific hybrid F1 of fraxinus. For. Eng. 29, 39–43. 10.16270/j.cnki.slgc.2013.05.040

[B13] Song L. Li D. Zeng X. Wu Y. Guo L. Zou Q. (2014). NDNA-PROT: identification of DNA-binding proteins based on unbalanced classification. BMC Bioinformatics 15:298. 10.1186/1471-2105-15-298 25196432 PMC4165999

[B14] Tan J. Li S. Zhang Z. Chen C. Chen W. Tang H.. (2019). Identification of hormone binding proteins based on machine learning methods. Math. Biosci. Eng. 16, 2466–2480. 10.3934/mbe.2019123 31137222

[B15] Tang H. Chen W. Lin H. (2016). Identification of immunoglobulins using Chou's pseudo amino acid composition with feature selection technique. Mol. Biosyst. 12, 1269–1275. 10.1039/C5MB00883B 26883492

[B16] Tang H. Zhao Y. Zou P. Zhang C. Chen R. Huang P.. (2018). HBPred: a tool to identify growth hormone-binding proteins. Int. J. Biol. Sci. 14, 957–964. 10.7150/ijbs.24174 29989085 PMC6036759

[B17] Theodoridis S. Koutroumbas K. (2009). Pattern Recognition, 4th Edn. Burlington, MA: Elsevier.

[B18] Wang W. Fang H. Groom L. Cheng A. Zhang W. Liu J.. (2008). Superoxide flashes in single mitochondria. Cell 134, 279–290. 10.1016/j.cell.2008.06.017 18662543 PMC2547996

[B19] Wei L. Tang J. Zou Q. (2017a). Local-DPP: an improved DNA-binding protein prediction method by exploring local evolutionary information. Inform. Sci. 384, 135–144. 10.1016/j.ins.2016.06.026

[B20] Wei L. Xing P. Shi G. Ji Z. Zou Q. (2019). Fast prediction of protein methylation sites using a sequence-based feature selection technique. IEEE/ACM Trans. Comput. Biol. Bioinformatics 16, 1264–1273. 10.1109/TCBB.2017.2670558 28222000

[B21] Wei L. Xing P. Su R. Shi G. Ma Z. S. Zou Q. (2017b). CPPred-RF: a sequence-based predictor for identifying cell-penetrating peptides and their uptake efficiency. J. Proteome Res. 16, 2044–2053. 10.1021/acs.jproteome.7b00019 28436664

[B22] Xu R. Zhou J. Liu B. Yao L. He Y. Zou Q. (2014). EnDNA-prot: identification of DNA-binding proteins by applying ensemble learning. Biomed. Res. Int. 2014:294279. 10.1155/2014/294279 24977146 PMC4058174

[B23] Zhang W. Liu J. Zhao M. Li Q. (2012). Predicting linear b-cell epitopes by using sequence-derived structural and physicochemical features. Int. J. Data Mining Bioinform. 6, 557–569. 10.1504/IJDMB.2012.049298 23155782

[B24] Zhao X. Jiao Q. Li H. Wu Y. Wang H. Huang S.. (2020). ECFS-DEA: an ensemble classifier-based feature selection for differential expression analysis on expression profiles. BMC Bioinformatics 21:43. 10.1186/s12859-020-3388-y 32024464 PMC7003361

